# Effect of Aminated Mesoporous Bioactive Glass Nanoparticles on the Differentiation of Dental Pulp Stem Cells

**DOI:** 10.1371/journal.pone.0150727

**Published:** 2016-03-14

**Authors:** Jung-Hwan Lee, Min-Sil Kang, Chinmaya Mahapatra, Hae-Won Kim

**Affiliations:** 1 Institute of Tissue Regeneration Engineering (ITREN), Dankook University, Cheonan 31116, Republic of Korea; 2 Department of Nanobiomedical Science and BK21 PLUS NBM Global Research, Center for Regenerative Medicine, Dankook University, Cheonan 31116, Republic of Korea; 3 Department of Biomaterials Science, College of Dentistry, Dankook University, Cheonan 31116, Republic of Korea; University of Helsinki, FINLAND

## Abstract

Mesoporous bioactive nanoparticles (MBNs) have been developed as promising additives to various types of bone or dentin regenerative material. However, biofunctionality of MBNs as dentin regenerative additive to dental materials have rarely been studied. We investigated the uptake efficiency of MBNs-NH_2_ with their endocytosis pathway and the role of MBNs-NH_2_ in odontogenic differentiation to clarify inherent biofunctionality. MBNs were fabricated by sol-gel synthesis, and 3% APTES was used to aminate these nanoparticles (MBNs-NH_2_) to reverse their charge from negative to positive. To characterize the MBNs-NH_2,_ TEM, XRD, FTIR, zeta(ξ)-potential measurements, and Brunauer–Emmett–Teller analysis were performed. After primary cultured rat dental pulp stem cells (rDPSCs) were incubated with various concentrations of MBNs-NH_2,_ stem cell viability (24 hours) with or without differentiated media, internalization of MBNs-NH_2_ in rDPSCs (~4 hours) via specific endocytosis pathway, intra or extracellular ion concentration and odontoblastic differentiation (~28 days) were investigated. Incubation with up to 50 μg/mL of MBNs-NH_2_ had no effect on rDPSCs viability with differentiated media (p>0.05). The internalization of MBNs-NH_2_ in rDPSCs was determined about 92% after 4 hours of incubation. Uptake was significantly decreased with ATP depletion and after 1 hour of pre-treatment with the inhibitor of macropinocytosis (p<0.05). There was significant increase of intracellular Ca and Si ion concentration in MBNs-NH_2_ treated cells compared to no-treated counterpart (p<0.05). The expression of odontogenic-related genes (BSP, COL1A, DMP-1, DSPP, and OCN) and the capacity for biomineralization (based on alkaline phosphatase activity and alizarin red staining) were significantly upregulated with MBNs-NH_2_. These results indicate that MBNs-NH_2_ induce odontogenic differentiation of rDPSCs and may serve as a potential dentin regenerative additive to dental material for promoting odontoblast differentiation.

## Introduction

Bioactive glass particles have been introduced as promising additives in the medical and dental fields, not only because of their apatite-forming, antibacterial, and neutralizing abilities, but also for their considerable mechanical properties and biofunctionality for hard tissue formation [1,2]. To date, these particles have been applied to various types of biomaterials, such as a bone or dentin scaffold matrix, dental composite resin, and regenerative endodontic materials [3–8]. Recently, bioactive glass nanoparticles have been developed that offer more surface area to combine with biomaterials and better biological and mechanical properties for substrate materials per weight of bioactive glass, as compared with conventional microsized bioactive glass [9–13].

Mesoporous material contains pores with diameters between 2 and 50 nm, intermediate in size between microporous (<2 nm) and macroporous (>50 nm) particles [14]. It has been suggested that mesoporous particles with well-ordered pores may act as potential vehicles for loading natural or synthetic biomolecules and orchestrating their release [15]. Although mesoporous silica was developed for biomedical uses, it has limited application for bone or dentin-pulp regeneration owing to its lack of bioactivity [16,17].

Mesoporous bioactive glasses have received considerable attention because they have highly ordered pores and greater bioactivity than conventional bioactive glasses [18]. Considering their desirable pore structure and superior *in vitro* bioactivity, mesoporous bioactive glasses may be promising biomaterials or additives for dental materials. Recently, mesoporous bioactive glass nanoparticles (MBNs) have been developed that combine the above-mentioned advantages of both nanoparticles and mesoporosity [19]. It has already been shown that the incorporation of MBNs in calcium phosphate cements improves bioactivity in simulated body fluid and that these nanoparticles can be used as vehicles to load and deliver therapeutic drugs or molecules [20–22]. Because most of these biomolecules and drugs have a negative charge [23,24], an amine group (−NH_2_) was introduced in the MBNs (MBNs–NH_2_) to change their naturally negative charge to a positive charge for loading drugs or biomolecules, and the uptake efficiency of nanoparticle is able to be increased owing to the attractive force between the negatively charged cells and MBNs-NH_2_ [22]. Therefore, such amination is one of the essential surface modifications that will allow these nanoparticles to interact with cells and exert biological effects, such as increased cell attachment and differentiation, and to combine with negatively charged therapeutic drugs or molecules [25,26].

Dentin–pulp regeneration using conventional dental materials is not easy because there is not enough bioactivity and cellular activity [27]. When dentin–pulp tissue is damaged, regenerated pulp tissue should be functionally competent, that is, capable of forming dentin to repair lost structure and generate dentin quickly to seal the clean pulp environment from the external oral environment [28]. Among the various promising bioactive materials developed thus far, MBNs—especially MBNs–NH_2_ that exhibit excellent bioactivity and cellular activity as a result of various released ions and their positive charge, or MBNs–NH_2_ incorporated in endodontic materials—are of great interest because of their potential use in regenerative endodontic applications [29,30]. Because MBNs–NH_2_ may possibly be detached from MBNs–NH_2_ incorporated in endodontic materials such as glass ionomer, calcium phosphate cement, and bonding agents and because MBNs themselves could be used as biofunctional material for regenerative endodontic medicine, the biological activity of MBNs–NH_2_ in dental pulp cells needs to be investigated.

Reports have shown that isolated dental pulp stem cells (DPSCs) can be induced to differentiate into odontoblast-like cells and produce dentin-like mineral structures *in vitro*, *and r*at dental pulp stem cells (rDPSCs) have been widely used in investigations of dentin–pulp regeneration owing to their high odontogenic potential and their easy accessibility, which allows them to be readily isolated from extracted rat teeth [31–33]. However, as far as we know, studies have not yet been carried out to determine whether MBNs–NH_2_ could promote odontoblast differentiation from rDPSCs or to assess the uptake of MBNs–NH_2_ via underlying endocytosis pathways.

The aim of this study was thus to investigate the uptake of MBNs–NH_2_ by rDPSCs and the endocytosis pathway involved, as well as the effects of MBNs–NH_2_ on odontogenic differentiation to clarify the inherent biofunctionality of MBNs–NH_2_.

## Materials and Methods

### Fabrication of MBNs-NH_2_

MBNs were prepared by sol-gel synthesis at room temperature in a mixed solution that included water, ethanol, and 2-ethoxyethanol as co-solvents; hexadecyltrimethylammonium bromide (CTAB) as a surfactant; aqueous ammonia as a catalyst; and calcium nitrate tetrahydrate as a precursor, according to procedures described elsewhere [34,35]. After the mixture was vigorously stirred (30 min), tetraethyl orthosilicate was added, and the solution was again vigorously stirred for 4 hours. The molar ratio of calcium oxide (CaO) to silicon dioxide (SiO_2_) was set at 15:85. A white precipitate was obtained in a gel state, filtered, washed with pure water, and dried in air at 60°C for 24 hours. The precipitate was then heated to 600°C at a rate of 1°C/min to remove any remaining CTAB, and the sample was finally calcined at 600°C in air for 5 hours. After washed with ethanol and de-ionized water (DW), nanoparticles were dried overnight in a vacuum to obtain MBNs. MBNs were then surface-functionalized with–NH_2_ groups through a post-synthesis procedure [23]. Briefly, 0.2% w/v of MBNs was dispersed in anhydrous toluene and refluxed (N_2_), with stirring, with 3% APTES at 80°C for 24 hours. The NH_2_-functionalized nanoparticles were then collected by centrifugation, washed with toluene, and finally dried at 80°C in a vacuum for 24 hours.

### Characterization of MBNs-NH_2_

The morphology and nanostructure of the samples were examined by means of high-resolution transmission electron microscopy (TEM) (JEM-3010, JEOL, Akishima, Tokyo, Japan) operating at 300 kV. To determine the glass phase of MBNs-NH_2_, X-ray diffraction (XRD) (Rigaku, Danvers, MA, USA) was used to scan specimens in the range of two-theta (2θ) diffraction angles, from 10 to 50 degrees, at a rate of 2 degrees/min, with a step-width of 0.02 degree using Cu Kα1 radiation at 40 kV and a current strength of 40 mA.

The chemical bond structures before and after amination were evaluated by means of diamond-crystal accessory attenuated total reflectance–Fourier transform infrared spectroscopy (ATR–FTIR) (PerkinElmer, Shelton, CT, USA) at a resolution of 4 cm^−1^ in the range of 4,000 to 400 cm^−1^.

The surface electrical properties of the MBNs-NH_2_ were investigated with zeta (ξ)-potential measurements (Zetasizer Nano ZS, Malvern Instruments, Malvern, UK), and the zeta-potential was measured in water at 25°C at a pH of 7 with an applied field strength of 20 V cm^−1^. This instrument automatically calculates the electrophoretic mobility (U), and the zeta-potential is obtained by means of the Helmholtz–Smoluchowski equation ξ = U*γ/ε, where ε is the di-electric constant and γ is the dispersing medium viscosity.

The mesoporous structure of MBNs-NH_2_ was analyzed according to N_2_ adsorption–desorption measurements using an automated surface area and pore size analyzer (Quadrasorb SI, Quantachrome Instruments, Boynton Beach, FL, USA). The specific surface area was determined according to the Brunauer–Emmett–Teller method, and the pore size distribution was assessed using the non-local density functional theory method.

For evaluating bioactivity, the *in vitro* apatite-forming ability of the samples was tested in Kokubo simulated body fluid at 37°C [36]. This fluid was prepared by dissolving NaCl (142.0 mM), KCl (5.0 mM), NaHCO_3_ (4.2 mM), CaCl_2_ (2.5 mM), MgCl_2_∙6H_2_O (1.5 mM), K_2_HPO_4_∙3H_2_O (1.0 mM), and Na_2_SO_4_ (0.5 mM) in DW adjusted to pH 7.4 with Tris-HCl buffer. After immersing MBNs-NH_2_ in the simulated body fluid for 14 or 28 days, XRD analysis was performed in the range of two-theta diffraction angles, from 5 to 70 degrees, at a rate of 2 degrees/min. The cumulative release of calcium and silicon ions from the particles was measured at 37°C for up to 28 days with inductively coupled plasma atomic emission spectrometry (ICP-AES) (Optima 4300DV, Perkin-Elmer, Waltham, MA, USA), according to the previous protocol [23]. Detection limit was determined as 0.1 ppm for Ca and Si ions. Three replicate samples were evaluated, and the mean ± standard deviation (SD) were recorded.

### Primary culture of rDPSCs

We obtained the rDPSCs from incisors isolated from 5-week-old male Sprague–Dawley rats. All animal protocols involving animals were approved by the Animal Care and Use Committee of Dankook University (DKU-12-015). After the rats were sacrificed, the rDPSCs collected from the upper and lower incisors were added to a phosphate balanced solution (PBS) (Gibco, Grand Island, NY, USA) containing 1% penicillin/streptomycin (Gibco). After the addition of 0.08% collagenase type I (Worthington Biochemical, Lakewood, NJ, USA), the solution was incubated for 30 minutes, with tapping every 10 minutes. Following enzyme digestion, the rDPSCs were recovered by centrifugation at 1,500 rpm for 3 minutes. The rDPSCs were cultured in α-MEM (LM 008–01, Welgene, Gyeongsan-si, Gyeongsangbuk-do, Korea) supplemented with 10% fetal bovine serum (FBS) (HyClone, Logan, UT, USA) and 1% penicillin/streptomycin (Gibco) at 37°C in a humidified atmosphere containing 5% CO_2_. Cells subcultured for three passages were used for whole experiments. To promote differentiation of the rDPSCs into odontoblasts, we cultured the cells in odontogenic supplemental medium (OM) that included 50 μg/mL of ascorbic acid, 100 nM of dexamethasone, and 10 mM of β-glycerophosphate, as described previously [37].

### Cell viability

After cells were plated at a volume of 100 μl of 10^5^ cells/mL per well on a 96-well plate (SPL Life Sciences, Pocheon-si, Gyeonggi-do, Korea) for 24 hours, ~40 μg MBNs-NH_2_ to 100 μl of 10^4^ rDPSCs (up to 400 μg MBNs-NH_2_ per mL of 10^5^ rDPSCs) was incubated in each well of a 96-well plate for further 24 hours with supplemented media or OM for undifferentiated or differentiated condition respectively. We assessed cell viability by means of a water-soluble tetrazolium salt (WST) assay at an absorbance of 450 nm using a plate reader (SpectraMax M2e, Molecular Devices, Sunnyvale, CA, USA) after washing and incubating for 1 hour with 10% WST salt (EA-Cytox, Daeil Lab, Seoul, Korea). To avoid interference by bioactive glass nanoparticle, 50 μl of supernatant was transferred to new 96-well plate and optical density was measured. Cell viability was determined based on the percentage of the optical density value for the experimental group over that of the control group. As control group, 100 μl of 10^4^ rDPSCs was only incubated with supplemented media or OM.

### Fabrication of fluorescent MBNs-NH_2_

To visualize the internalization (uptake) of MBNs-NH_2_ into the rDPSCs, we prepared fluorescein isothiocyanate isomer I (FITC)-conjugated MBNs-NH_2_ according to the below protocols. 0.1% w/v of FITC and MBNs-NH_2_ were mixed together in dimethylformamide and incubated overnight. Conjugated particles were suspended in distilled water after being washed three times with ethanol at 10,000 rpm for 3 minutes each time. FITC-conjugated MBNs-NH_2_ (1 μg) were incubated with 2×10^4^ rDPSCs (at a concentration of 5 μg/mL of 10^5^ rDPSCs) in each well of a 24-well plate for a pre-determined period of time and were fixed with 10% formaldehyde (Junsei Chemical, Chuo-ku, Japan). The concentration was decreased to one tenth of previous 50 μg/mL of nanoparticle to 10^5^ rDPSCs to minimize overlapping cells and internalized nanoparticle.

The samples were then subjected to regular immunocytochemical procedures; treated with phalloidin (1:40) (A34055, Alexa Fluor 555 Phalloidin, Invitrogen, Life Technologies, Grand Island, NY, USA) for cytoskeletal F-actin staining at room temperature for 20 minutes; and counterstained with 4′,6-diamidino-2-phenylindole (DAPI) (1:200,000) (D1306, Invitrogen) to observe the nucleus by means of a confocal laser scanning microscope (LSM 510, Zeiss, Switzerland). The internalization of MBNs-NH_2_ in the cells was observed by Z-stack imaging. FITC-conjugated MBNs-NH_2_, F-actin, and nuclei were stained green, red, and blue, respectively.

### Quantitative analysis of internalization of MBNs-NH_2_ using flow cytometry

To quantify the internalization of MBNs-NH_2_ into the cells and to determine the pathway of internalization, 150 μg of FITC-conjugated MBNs-NH_2_ was incubated with 3×10^5^ rDPSCs in each well of a 6-well plate (50 μg per mL of 10^5^ rDPSCs) for a pre-determined period of time (0.25, 0.5, 1, 2, 4 hours). To determine the pathway of internalization, we pre-incubated rDPSCs with 1 mM of amantadine hydrochloride (A1260, Sigma, St. Louis, MO, USA), 100 mM of genistein (G6649, Sigma), and 2.5 mM of 5-(N-ethyl-N-isopropyl) amiloride (A3085, Sigma) for 1 hour before treating the FITC-conjugated MBNs-NH_2_. To determine whether the endocytosis was ATP-dependent, the cells were incubated at 4°C for 4 hours or were pre-treated with 100 mM of sodium azide for 1 hour. After incubating with various concentration of each chemical for 6 hours, maximum concertation which showed non-cytotoxicity to rDPSCs was chosen to pretreat the cells. After trypsinization (Gibco), the cells were centrifuged at 10,000 rpm for 5 minutes, and the pellets were fixed with ice-cold PBS. Subsequently, 400 μL of binding buffer was added to each tube prior to analysis with a FACSCalibur flow cytometer (BD Biosciences, San Jose, CA, USA). Data for 10,000 cells in each sample were analyzed using the CellQuest Pro software (v.5.1 BD Biosciences).

### Internalization of MBNs-NH_2_ on TEM

TEM was used to observe internalization of the MBNs-NH_2_. After 4 hours of incubation with MBNs-NH_2_ in a particle:cell ratio of 50 μg/mL of 10^5^ rDPSCs, the rDPSCs were fixed for 12 hours in 2% glutaraldehyde (Merck, Kenilworth, NJ, USA) and paraformaldehyde (Merck) in PBS (Tech&innovation, Chuncheon, Gangwon, Korea), pH 7.4. They were then post-fixed with 1% osmium tetroxide (Polysciences, Warrington, PA, USA), dissolved in PBS for 2 hours, and dehydrated in a gradually ascending series (from 50 to 100%) of ethanol (Merck) and infiltrated with propylene oxide. Specimens were embedded using the Poly/Bed 812 Embedding Kit (Polysciences) according to the manufacturer’s protocol. Sections 200 to 250 nm thick were initially prepared and stained with toluidine blue (Sigma) for observation under a light microscope. Sections of 70-nm thickness were double-stained with 6% uranyl acetate (Electron Microscopy Sciences, Hatfield, PA, USA) and lead citrate (Fisher, Carlsbad, CA, USA) for 20 minutes and 10 minutes, respectively. Stained sections were cut by Leica EM UC7 (Leica Microsystems, Vienna, Austria) with a diamond knife (Diatome) and transferred onto copper and nickel grids. All the sections were observed on TEM (JEM-1011, JEOL) at the acceleration voltage of 80 kV.

### Detection of calcium and silicon ions

Calcium and silicon ions from cells and odontoblastic differentiated media from rDPSCs not treated or treated with MBNs–NH_2_ were detected at 4, 12, 24, 48, or 72 hours incubation time. After 150 μg of MBNs–NH_2_ was incubated with 3×10^5^ rDPSCs in 3 mL in each well of a six-well plate (50 μg per mL of 10^5^ rDPSCs) for a pre-determined period of time (4, 12, 24, 48, or 72 hours), cultured media and cell pellets were gathered for observation using inductively coupled plasma atomic emission spectrometry (ICP-AES) (Optima 4300 DV, PerkinElmer). The cultured media were centrifuged at 12,000 rpm for 10 minutes and 1.5 mL of supernatant was gently obtained to remove any remaining nanoparticles. For detection of ion concentration in the cells, the cells were sequentially washed three times with PBS, were trypsinized, and underwent centrifugation (2,000 rpm, 3 min) to remove noninternalized nanoparticles. After removing supernatant, cell pellets were dried, and 9 mL of 70% of nitric acid (Sigma) was added. After these mixtures were incubated on a hot plate for 3 hours at 180°C and then cooled to room temperature, 15 mL of DW was added and volatilized. The final volume was set at 1.5 mL with DW, which was the same volume as the cultured media so the ion concentrations between the cells and the media could be compared. ICP-AES was used to detect the total Ca and Si ion concentrations with argon plasma (6000 K). The detection limit for the Ca and Si ions was determined to be 0.1 ppm. According to the preliminary results, there were no statistical differences in cell numbers at 4, 12, 24, 48, or 72 hours of incubation under OM with or without MBNs–NH_2_ (data not shown).

### mRNA expression

To measure mRNA expression, we incubated 45 or 90 μg/mL of MBNs-NH_2_ in 6×10^4^ rDPSCs (at a concentration of 25 or 50 μg/mL of 10^5^ rDPSCs) in each well of a 24–well plate for 3, 7, 14, and 21 days with OM. The rDPSCs were cultured with or without OM as controls. Total RNA was extracted from the rDPSCs using Ribospin (GeneAll, Seoul, Korea), and 1 μg was reverse-transcribed to cDNA using oligo-dT primer (Venlo, Netherlands, Qiagen), a pre-mixture for reverse transcription (AccuPower RT PreMix, Bioneer, Seongnamsi, Gyeonggi-do, Korea), and a 2720 Thermal Cycler (Applied Biosystems, Foster City, CA, USA). Quantitative real-time PCR experiments were performed using a Power SYBR Green PCR Master Mix (Applied Biosystems) and real-time PCR equipment (StepOnePlus, Applied Biosystems) according to the manufacturer’s instructions. Real-time PCR was performed using the following primer sequences with four sample (n = 4) in each condition: bone sialoprotein (BSP), forward 5′-ACAGCTGACGCGGGAAAGTTG-3′ reverse 5′-ACCTGCTCATTTTCATCCACTTC-3′; collagen type I alpha 1 (COL1A), forward 5′-CGTGACCAAAAACCAAAAGTGC-3′ reverse 5′-GGGTGGAGAAAGGAACAGAAA-3′; dentin matrix protein 1 (DMP1), forward 5′-GGACGGCTCTGAGTTCGA-3′ reverse 5′-TGGGTTTCCCTGCTGTTG-3′; dentin sialophosphoprotein (DSPP), forward 5′-TGGAGACGCCACCCTTGT-3′ reverse 5′-TCTATCCCGTTCCCGCTA-3′; and osteocalcin (OCN), forward 5′-AGACTCCGGCGCTACCTCAACAAT-3′ reverse 5′-CAGCTGTGCCGTCCATACT-3′. After confirming qPCR efficiency from no template controls, positive controls (OM treatment) and no amplification controls, mRNA expression levels of sample (n = 4) in each condition were normalized to house keeping gene (GAPDH) and relative fold change in expression was automatically calculated using 2^-ΔΔCt^ value with respect to undifferentiated rDPSCs by StepOne software v2.3.

### Alkaline phosphatase assay

To measure ALP activity, we incubated 45 or 90 μg/mL of MBNs-NH_2_ in 6×10^4^ rDPSCs (at a concentration of 25 or 50 μg/mL of 10^5^ rDPSCs) in each well of a 24–well plate for 7, 14, and 21 days with OM. At days 7, 14, and 21, the media were removed and 1 mL of 0.2% Triton X-100 (Sigma) was added to each well. After cell lysates were placed in a 1.5-mL centrifuge tube, and the samples were processed through three freeze–thaw cycles (−70°C and room temperature, 45 minutes each) to rupture the cell membranes and extract the proteins and DNA from the cells, which were then centrifuged for 20 minutes at 13,000 g at 4°C. From the supernatant, alkaline phosphatase (ALP) activity (n = 5) was measured using p-nitrophenyl phosphate (3.3 mM final concentration) as the substrate in 0.25 M of 2-aminomethyl-1-propanol (pH 10.3) and 0.16 mM of MgCl_2_. Absorbance was measured at a wavelength of 405 nm using a plate reader and was normalized to the dsDNA quantity measured by the PicoGreen assay (n = 5), and dsDNA from the supernatant was quantified using the Quant-iT PicoGreen Kit (Invitrogen) following standard protocols. Briefly, 100 μL of each cell lysate solution was added to 100 μL of PicoGreen reagent and incubated in the dark at room temperature for 5 minutes. The absorbance was read at an excitation/emission of 480/520 nm on the plate reader.

### Alizarin red staining

To perform ARS staining, we incubated 45 or 90 μg/mL of MBNs-NH_2_ in 6×10^4^ rDPSCs (at a concentration of 25 or 50 μg/mL of 10^5^ rDPSCs) in each well of a 24–well plate for 28 days with or without OM. The rDPSCs were fixed in 10% formaldehyde for 30 minutes and rinsed with distilled water. Cells were stained with 40 mM of alizarin red S (pH 4.2) for 30 minutes, with gentle agitation. The level of alizarin red staining (ARS) was observed under light microscopy. To quantify the staining, 10% cetylpyridinium chloride (Sigma) in 10 mM of sodium phosphate, pH7.0, was added to the culture dish for de-staining, and the culture dish was incubated for 30 minutes at room temperature. ARS concentration was determined by an absorbance measurement at 562 nm on a multiplate reader.

### Statistical analysis

All data are reported as the mean ± SD and images are shown after at least triplicate independent experiment sets. Statistical analysis was carried out using one-way analysis of variance (ANOVA), followed by a Tukey’s post hoc test, where significance was declared at p < 0.05.

## Results

### Characterization of MBNs-NH_2_

**Fig 1:**
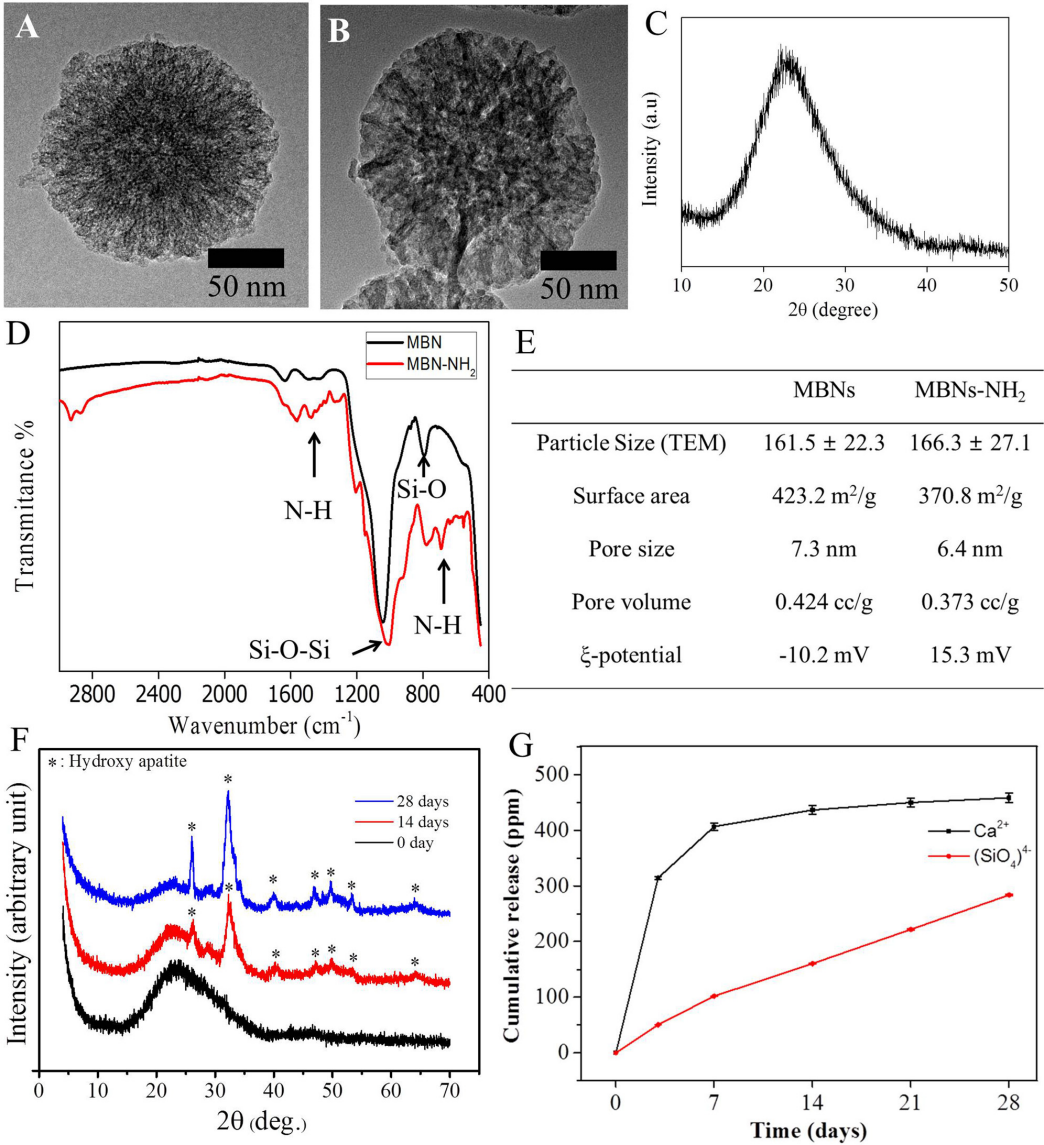
Characteristics of MBNs before and after amination_._ Transmission electron microscopic images showing MBNs (a) and MBNs-NH_2_ (b) with a highly mesoporous structure. (**c**) X-ray diffraction (XRD) pattern of MBNs-NH_2_ showing an amorphous glass phase. (**d**) Amine functionalization was confirmed on Fourier transform infrared spectroscopy (FTIR). (**e**) A shift of zeta (ξ)-potential to positive charge was observed after amine functionalization and summary of mesoporous characteristics of MBNs and MBNs-NH_2_. (**f**) XRD pattern of MBNs-NH_2_ after immersion in simulated body fluid for 14 or 28 days showing hydroxyapatite precipitation. (**g**) Cumulative release of ions from MBNs-NH_2_ detected by inductively coupled plasma atomic emission spectrometry (ICP-AES) analysis. The measurements were performed in triplicate, and representative results are shown.

The typical spherical nanoparticles (≈160 nm) of monodispersed sizes were observed in TEM images before and after amination (Fig 1A and 1B). XRD patterns of the MBNs revealed a broad, amorphous pattern from 15 to 30 degrees of two-theta diffraction angles (Fig 1C). FTIR spectra of MBNs-NH_2_ showed additional bands (N–H at 695 cm^−1^ vibrations and 1,570 cm^−1^ bending mode), as compared with those of non-aminated MBNs (Fig 1D). MBNs before amination showed a negative zeta-potential (−10.2 mV), whereas APTES treatment for amination changed the zeta-potential values to positive values (+15.3 mV) (Fig 1E).

Characteristics of the MBNs-NH_2_ were evaluated by the N_2_ adsorption–desorption measurement and are summarized in Fig 1E. The N_2_ adsorption–desorption isotherms (Figure A in S1 File) exhibited characteristics of the type IV isotherm of mesoporous materials according to the IUPAC classification [38]. MBNs showed high mesoporosity, including a specific surface area of 423.8 m^2^g^−1^ and a pore volume of 0.424 cm^3^g^-1^. The size of the mesopores was determined to be 7.3 nm. After amination, the surface area was 370.8 m^2^g^−1^ and pore volume was 0.373 cm^3^g^−1^ with 6.4 nm of pore size_._

The bioactive (apatite-forming) ability of the MBNs-NH_2_ in simulated body fluid was also assessed. After immersion in simulated body fluid for 14 or 28 days, apatite peaks (including a main peak at 2θ~32) had developed in MBNs-NH_2_ and peaks have higher intensity in 28 days than 14 days, as examined by XRD (Fig 1F). The cumulative release profiles of Si and Ca ions from the mesoporous nanoparticles were monitored by ICP-AES for periods of up to 14 days, as presented in Fig 1G. Over the test period, the release of Ca ions was observed to be higher than that of Si ions. Although the amount of Ca ions released was characterized by a large initial burst (314 and 407 ppm on 3 and 7 days) followed by trivial changes over the test period (436, 450, and 458 ppm on days 14, 21, and 28, respectively), the amount of released Si ions increased over time (50, 102, 160, 222, and 284 ppm on days 3, 7, 14, 21, and 28, respectively). Ca ions released faster than Si ions along with the other mesoporous bioactive glass particles [39,40].

### Cell viability and internalization of MBNs-NH2

**Fig 2:**
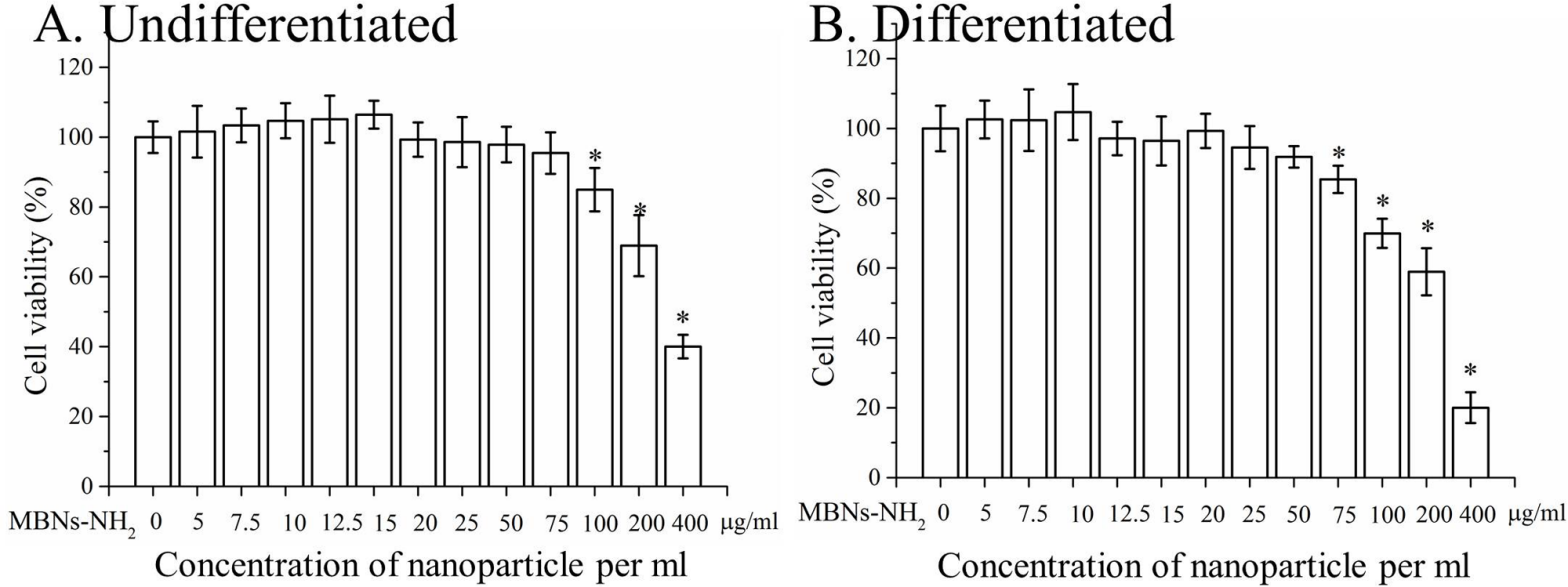
Cell viability after 24 h incubation with various concentrations of MBNs–NH_2_ in (a) undifferentiated or (b) differentiated rDPSCs. After cells were plated at a volume of 100 μL of 10^5^ cells per well on a 96-well plate for 24 h, 5, 7.5, 10, 12.5, 15, 20, 25, 50, 75, 100, 200, or 400 μg MBNs–NH_2_ per mL of initial 10^5^ rDPSCs (0.5, 0.75, 1, 1.25, 1.5, 2, 2.5, 5, 7.5, 10, 20, or 40 μg MBNs–NH_2_ to 100 μL of 10^4^ rDPSCs) was incubated in each well of a 96-well plate for an additional 24 hours with conventional supplemented media or odontogenic supplemental media. After 1 day, cell viability was determined using water-soluble tetrazolium based on the percentage of the optical density (450 nm) value in the experimental group versus the non-treated control. Assays were independently performed in triplicate. Representative averages (n = 5) and SD were recorded. * Significant difference vs. non-treated control (p<0.05).

**Fig 3:**
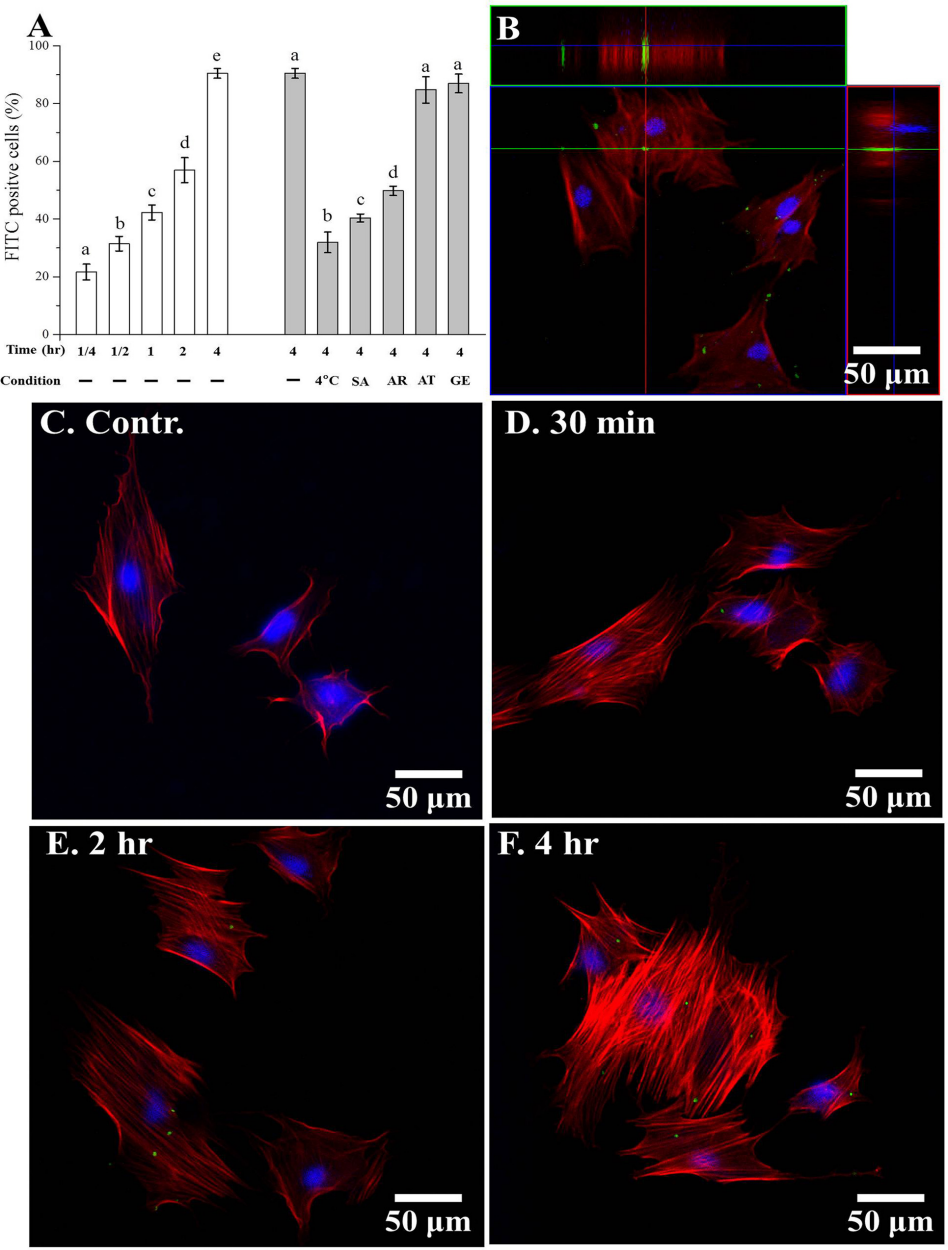
Uptake of MBNs–NH_2_ into rDPSCs within 4 h with or without pre-treatment using various types of inhibitors or culture conditions. Incubation at 4°C was used to prevent ATP-dependent endocytosis. (**a**) Uptake of FITC-labeled MBNs–NH_2_ into rDPSCs was characterized by flow cytometry depending on incubating time and on pre-treatment condition (1 h), which included sodium azide (SA) (100 mM), 5-(N-ethyl-N-isopropyl) amiloride (AR) (2.5 mM), amantadine-HCl (AT) (1 mM), and genistein (GE) (100 mM). Different letters indicate significant differences at p<0.05. Confocal images of rDPSCs incubated with MBNs–NH_2_. (**b**) 3D reconstructions and views of the xz- and yz-planes showing the FITC-labeled MBNs–NH_2_ (green) internalized by the cells and associated with the actin cytoskeleton (red). (**c—f**) Depending on the incubation time, increases in FITC-labeled MBNs–NH_2_ (green) were detected in the rDPSCs (Red = F-actin filaments, blue = nucleus, and green = FITC-labeled MBNs–NH_2_.) The measurements were performed in triplicate, and representative data or images are shown.

To investigate the biocompatibility of MBNs-NH_2_, cell viability was measured using rDPSCs co-incubated with various concentrations of MBNs-NH_2_ in OM for 24 hours. Viability was significantly lower than in the no-treatment group when the MBNs-NH_2_ concentration exceeded 100 μg/mL in undifferentiated rDPSCs (Fig 2A) while cytotoxicity was observed over 75 μg/mL in differentiated rDPSCs (p<0.05) (Fig 2B). To evaluate uptake of MBNs-NH_2_ by rDPSCs, the number of cells that took up FITC-conjugated MBNs-NH_2_ was detected by green fluorescence. Only 15 minutes of incubation with MBNs-NH_2_ revealed a ≈20% uptake in rDPSCs, and this efficiency increased up to 92% after 4 hours of incubation (Fig 3A).

**Fig 4:**
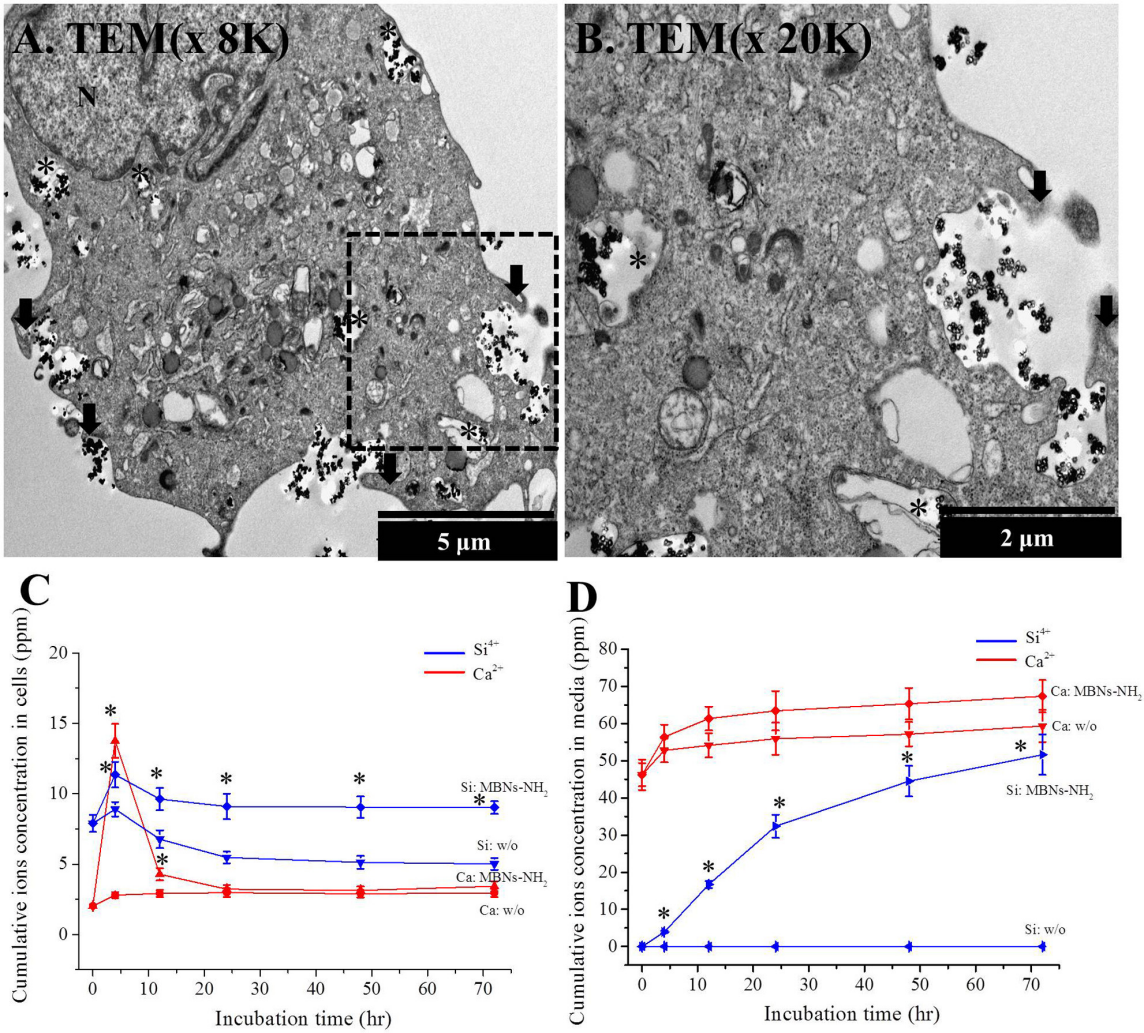
(a, b) Macropinocytosis of MBNs–NH_2_ into rDPSCs after 4 hours of incubation by TEM images and Ca and Si ion concentrations from total cells (c) or cultured odontogenic supplemental media (OM) (d) by ICP-AES. Low-magnification (a, ×8,000) and high-magnification (b, ×20,000, black dot box from (a)) TEM images of rDPSCs after treatment with MBNs–NH_2_ (50 μg/mL of 10^5^ cells) for 4 hours. Macropinocytosis with membrane ruffles was only observed without clathrin or caveolae-coated membrane or vesicle. Black arrow shows membrane ruffles, a typical characteristic of macropinocytosis; black asterisks indicate the intracellular distribution of MBNs–NH_2_ in endosomes. Significant increases in Si ion concentration from total cells were detected at 12, 24, 48, and 72 hours in the MBNs–NH_2_–treated rDPSCs compared to control (without [w/o] treatment), while Ca ion concentrations were significantly detected at 4 and 12 h of incubation (p<0.05). According to the preliminary results for cell numbers at 4, 12, 24, 48, or 72 hours of incubation under OM with or without MBNs–NH_2,_ there was no significant difference. * Statistically significant difference in MBNs–NH_2_–treated group compared with the untreated group (p<0.05).

To determine the mechanism underlying the uptake of MBNs-NH_2_, the following experiments were carried out. First, to confirm uptake of MBNs-NH_2_ by ATP-dependent endocyotosis, the culture temperature was decreased to 4°C and incubated for 4 hours or the cells were treated with 100 mM of sodium azide for 1 hour, which showed significant decrease of uptake of MBNs-NH_2_ (Fig 3A and Figure B in S1 File). To investigate which ATP-dependent endocytosis pathway is involved, various pretreatment was performed in Fig 3A. Uptake of MBNs-NH_2_ was significantly blocked by amiloride (p<0.05), whereas amantadine and genistein did not appear to influence uptake (p>0.05, Fig 3A and Figure B in S1 File). Fig 3B through 3F show that, after incubation with MBNs-NH_2_ for 30 minutes, 2 hours, and 4 hours, green particles could be seen dispersed in the cytosol, overlapping with a red cytoskeletal structure. Z-stack images after 4 hours of incubation showed internalization of green-stained MBNs-NH_2_ (Fig 3B), which was confirmed on TEM images (Fig 4A and 4B). The black asterisks indicate the intracellular distribution of MBNs-NH_2_ in endosomes, and the black arrows show engulfing MBNs-NH_2_ and the surrounding fluid of the extracellular fluid with membrane ruffles, which is typical characteristic of macropinocytosis (Fig 3G and 3H). In addition, TEM images did not show any clathrin or caveolin coated vesicle and pit. The number of green particles included cells was greater at 4 hours of incubation than at 30 minutes or 2 hours (Fig 3C through 3F). Ca and Si ion concentration from total cells or cultured media by ICP-AES is shown (Fig 4C and 4D). Significant increase of intracellular Si ion concentration was detected at 4, 12, 24, 48 and 72 hours in MBNs-NH_2_ treated rDPSCs compared to without (w/o) treatment control (p<0.05), while Ca ion concentration was significantly detected in 4 and 12 hours (p<0.05). According to the preliminary results about cell numbers at 4, 12, 24, 48 or 72 hours incubation under OM with or without MBNs-NH_2,_ there was no statistical difference. Significant increase of Si ion concentration was detected at 4, 12, 24, 48 and 72 hours in MBNs-NH_2_ treated media compared to without (w/o) treatment control (p<0.05).

### Differentiation of rDPSCs into odontoblast

**Fig 5:**
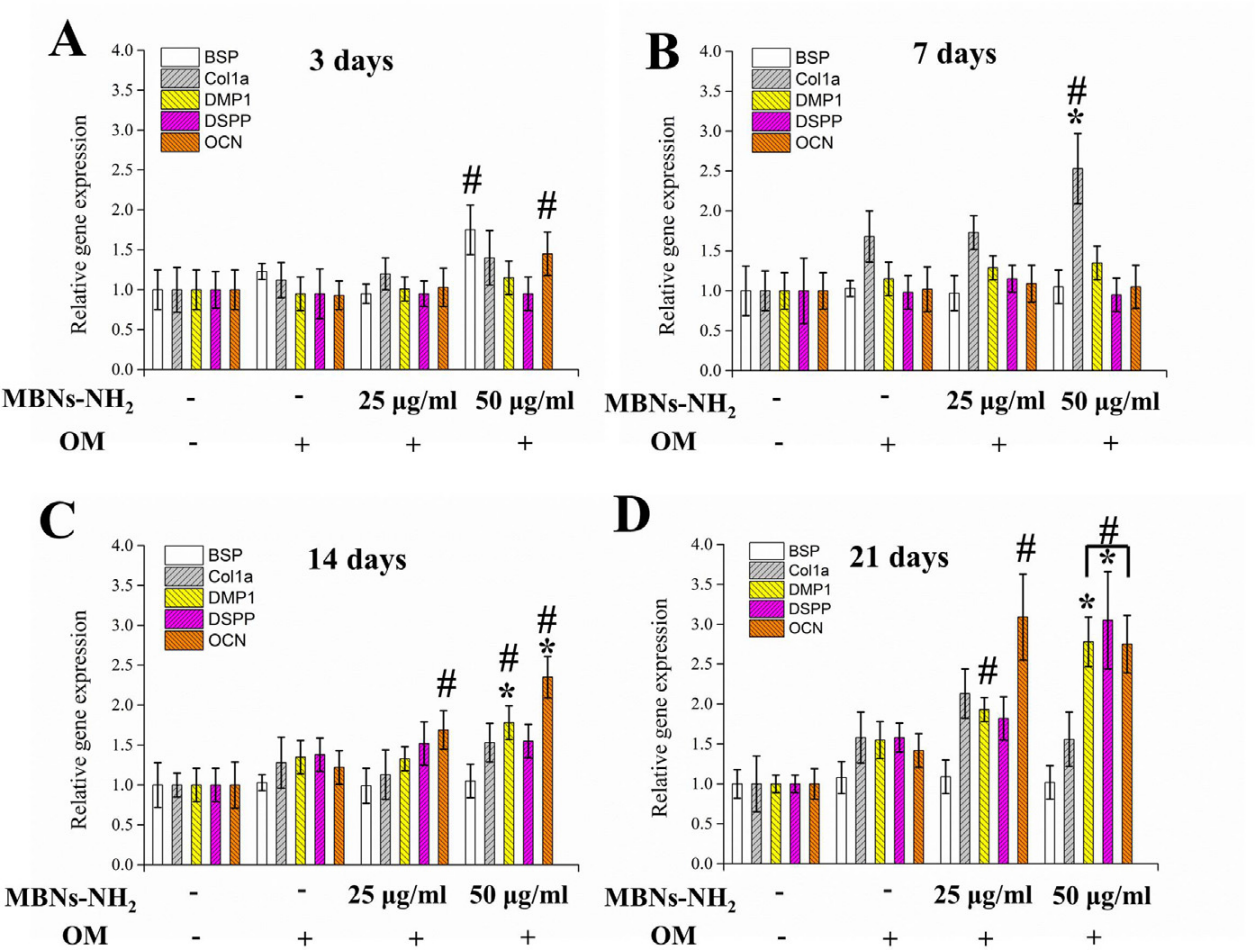
Effects of MBNs-NH_2_ in rDPSCs on mRNA expression of odontoblasts at 3 days (**a**), 7 days (**b**), 14 days (**c**), and 21 days (**d**) of incubation with odontogenic supplemental medium (OM). # Statistically significant difference vs. OM group (p<0.05). * Statistically significant difference between MBNs-NH_2_ –treated groups (p<0.05). The measurements were performed in triplicate, and representative average (n = 5) are shown with SD.

**Fig 6:**
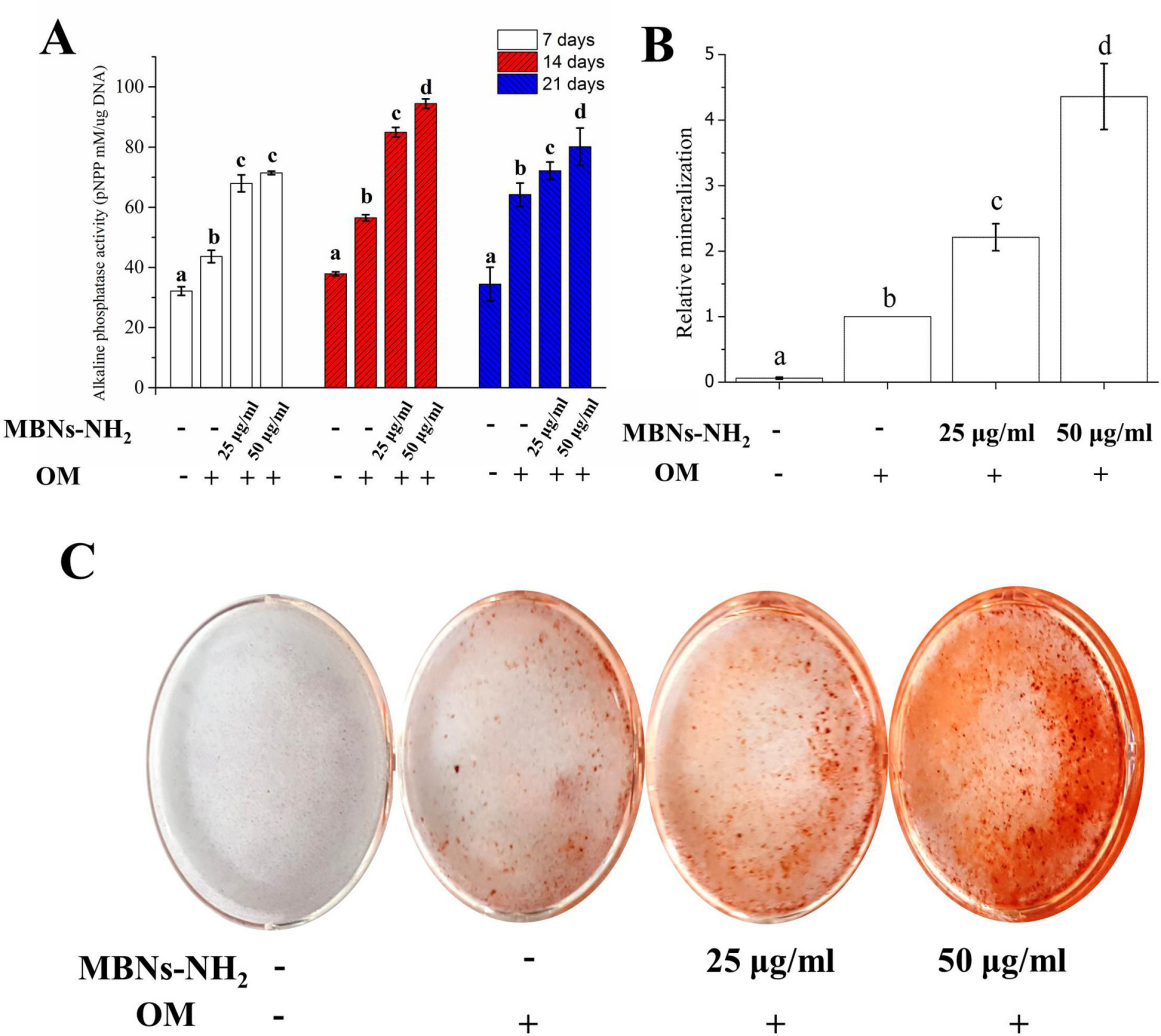
Effects of MBNs-NH_2_ in rDPSCs on alkaline phosphatase (ALP) activity (at 7, 14, and 21 days) (**a**) and on calcium deposition using alizarin red staining (ARS) (at 28 days) (**b** and **c**). After ARS was observed under the microscope (**c**), 10% w/v of cetylpyridinium chloride monohydrate was used for quantitative analysis (**b**). Cells were incubated for 7 to 28 days in odontogenic supplemental medium (OM). Different letters indicate significant differences among them at p<0.05. The measurements were performed in triplicate, and representative average (n = 5) with SD or images are shown.

Effects of MBNs-NH_2_ internalization on differentiation of rDPSCs, gene expressions of odontoblast markers, ALP activity, and *in vitro* calcification were investigated with OM. As demonstrated in Fig 5A and 5B, after 3 and 7 days of culture, mRNA expression of BSP, COL1A, or OCN genes was significantly increased in the cells treated with 50 μg/mL of MBNs-NH_2,_ as compared with the OM-treated control (p<0.05). After 14 and 21 days, mRNA levels of DMP1, DSPP, and OCN were upregulated in the MBNs-NH_2–_treated cells to a greater extent than in the OM-treated control cells (Fig 5C and 5D, p<0.05). At 21 days of incubation, mRNA levels of DMP1 and OCN were significantly increased in the cells treated with 50 μg/mL of MBNs-NH_2_, as compared with those treated with 25 μg/mL of MBNs-NH_2_. At 7, 14, and 21 days of culture, statistically significant increases in ALP activity were noted in the MBNs-NH_2_–treated cells, as compared with the untreated or OM-treated control cells (Fig 6A). Moreover, there was a clear difference between the MBNs-NH_2_–treated cells and the untreated or OM-treated control cells on the ARS-stained images, and quantification of the eluted products revealed a statistically significant difference between the MBNs-NH_2_ groups (p<0.05) (Fig 6B and 6C).

## Discussion

MBNs–NH_2_ have been shown to play a vital role as an additive in the successful regeneration of the dentin–pulp complex or bone tissues when used in fabricating three-dimensional scaffolds [9,21,34]. MBNs–NH_2_ not only increase mechanical or biological properties but also can act as vehicles for growth factors, therapeutic drugs, or biomolecules (e.g., siRNA, recombinant protein) [34]. Because MBNs–NH_2_ may possibly be released from the three-dimensional scaffold or other dental materials such as glass ionomer, calcium phosphate cement, and bonding agents, and because the MBNs themselves could be used as biomolecules for regeneration in dentistry, investigations of the biological activity of MBNs–NH_2_ in mammalian cells are needed. After characterizing MBNs–NH_2_ as compared with MBNs, we investigated the internalization of MBNs–NH_2_ in rDPSCs and their potency of differentiation into odontoblasts to determine their potential as dentin–pulp regenerative additives.

### Characterization of MBNs–NH2

First, MBNs and MBNs–NH_2_ were fabricated in nanosize (≈160 nm) and were found to have well-ordered mesoporous characteristics (≈7 nm) (Fig 1A and 1B). XRD data showed a typical amorphous glass structure, which is one of the key characteristics for the bioactivity of bioactive glass (Fig 1C). Even though MBNs have beneficial characteristics, such as increased surface area, porosity, and mesopores, and the ability to load biomolecules or therapeutic drugs, as compared with conventional bioactive glass nanoparticles, the naturally negative charge of MBNs is a major hurdle in attempts to load negatively charged biomolecules or therapeutic drugs [41]. To change the surface charge from negative to positive, MBNs must be treated with the proper concentration of APTES (to form MBNs–NH_2_). This process is frequently used for amine-functionalizing in the manufacture of plastic products, which results in a change in the surface zeta-potential from a negative value (−10.2 mV) to a positive value (+15.3 mV) (Fig 1E). This is the result of a change in the surface chemical status from a hydroxyl to an amino group, which has been confirmed by FTIR analysis (Fig 1D)[23].

After amination, the physical characteristics of MBNs–NH_2_, including particle size, surface area, pore size, and pore volume, were changed less than 15% (Fig 1E). The bioactivity of MBNs–NH_2_ might be questioned, and their ability to form apatite crystals in simulated body fluid was investigated (Fig 1F). Our results revealed clear hydroxyapatite formation, as has been reported previously [20–23,34]. In addition, therapeutic Ca and Si ions were released for up to 28 days, with approximately 16 ppm of Ca ions and 10 ppm of Si ions being released from 20 mg of MBNs–NH_2_ per day. It is well known that these ions induce odontoblast differentiation (Fig 1G) [9,42]. Therefore, thanks to their ability to induce apatite mineralization and release therapeutic ions, MBNs–NH_2_ are considered to be useful dentin-regenerative nano-additives when used alone or when incorporated within biopolymer matrices, calcium phosphate cement, and dentin bonding agents [20–22,34].

### Internalization of MBNs–NH2 in rDPSCs

To investigate the direct effect of MBNs–NH_2_ in terms of odontoblast differentiation, we conducted a cell viability analysis. Along with the other results, the differentiated cells had more cytotoxic sensitivity to MBNs–NH_2_ than did the undifferentiated cells [43,44]. The treatment of rDPSCs with up to 50 μg/mL of MBNs–NH_2_ resulted in no change in differentiated cell viability; consequently, we used less than 50 μg/mL for further assays.

It is known that nanoparticles can be transported into cells through several different pathways, such as nonspecific diffusion or ATP-dependent endocytosis (uptake), which encompasses the process of membrane manipulation to envelop and absorb materials [45]. ATP-dependent endocytosis for nanoparticles takes three forms: macropinocytosis, receptor-mediated endocytosis (clathrin-mediated or caveolae-mediated), and phagocytosis, which mostly takes place in macrophages. First, the uptake efficiency of MBNs–NH_2_ increased from 23% within 15 minutes to 92% within 4 hours. To reveal the mechanism underlying the uptake of MBNs–NH_2_ in rDPSCs, two experiments were carried out: cell culture at 4°C with MBNs–NH_2_ and pre-treatment with 100 mM of sodium azide prior to MBNs–NH_2_ co-incubation, both of which induced cellular ATP depletion [46].

After confirming the critical role of ATP in inducing endocytosis, and considering the cell type of rDPSCs, specific pathways were determined by 1 hour of pre-treatment with 5-(N-ethyl-N-isopropyl) amiloride, amantadine-HCl, and genistein added to the rDPSCs, which act as inhibitors of macropinocytosis, clathrin-mediated endocytosis, and caveolae-mediated endocytosis, respectively, and macropinocytosis was considered to have a major role in endocytosis [47]. Pretreatment with drugs to block specific endocytosis pathways has been widely used to determine the major endocytosis pathway in biological and nanoparticle research [47–50]. However, some researchers were uncertain about using drugs for blocking distinct endocytic pathways owing to poor specificity, cell line–dependent inhibitory effects, and cytotoxicity [51,52]. To reduce the cytotoxic influence on cellular uptake, *in vitro* cytotoxicity was investigated with various concentrations of each blocking drug, and a non-cytotoxic maximum concentration was chosen for treating the rDPSCs. Although we did not include controls for evaluating the endocytosis-blocking specificity of each drug to the rDPSCs in this experiment, we chose 5-(N-ethyl-N-isopropyl) amiloride, amantadine-HCl, and genistein as specific inhibitors of macropinocytosis, clathrin-mediated endocytosis, and caveolae-dependent endocytosis, respectively, because their use has proved to be promising [53,54]. Phagocytosis involves the ingestion of materials up to 10 μm in diameter [55] and can be accomplished by a few cell types, such as macrophages, neutrophils, and dendritic cells. On the other hand, micropinocytosis, clathrin-mediated, and caveolae-mediated endocytosis are uptake mechanisms that can be carried out by virtually all cell types, and they normally involve the ingestion of nanosized or sub-micron-sized material in solution.

TEM studies have suggested that macropinocytosis in the cellular uptake of MBNs–NH_2_ occurs via the plasma membrane, which has a structure different from that of other endosomes consisting of clathrin or caveolae-coated membrane and from the morphology of the lysosomal vesicle [56–58]. After combining results from the inhibitory drug study and the typical characteristic of macropinocytosis (membrane ruffles) noted on TEM analysis, for the MBNs–NH_2_ particles, we determined that macropinocytosis played a major role; the caveolae-mediated and clathrin-mediated endocytosis uptake pathways did not contribute. Macropinocytosis is the major form of nonspecific endocytosis responsible for the majority of cases of fluid-phase uptake for a number of cell types [48] and is reportedly involved in the uptake of nanoparticles such as silica nanoparticles (80~500 nm), aminated silica nanoparticles (~300 nm), and non-aminated bioactive glass nanoparticles (215±20 nm) [47,59,60].

The intracellular localization route consists of endosome, early lysosome, and late lysosome, which are basic intracellular pathways of nanoparticles; therefore, both endosomes and lysosomes are confined as subcellular organelles [61]. The formation of endosomes for up to 4 hours is considered to be biocompatible and a stabilizing characteristic of nanoparticles inside cytoplasm for drug delivery, whereas fusion into the lysosomal vesicle is regarded as an inappropriate characteristic owing to rapid degradation of the loaded drug and exocytosis of the nanoparticles [62,63]. Therefore, the fabricated MBNs–NH_2_ used in our study are also assumed to have successful biocompatibility as carriers for therapeutic drugs or biomolecules and can be used as a safe additive for prolonged drug delivery in dental materials without severe impairment to the intracellular organisms [60]. In addition, MBNs–NH_2_ were found to involve ATP-dependent macropinocytosis pathway, which is considered to be a more biocompatible pathway than non-compatible ATP-independent endocytosis [64,65].

When bioactive glass nanoparticles pass through the cell membrane, they show a reduction in size, suggesting that the particles may have dissolved inside the cells and released ions [59]. According to the previous experiment, MBNs–NH_2_ released 0.49 μM/μg·day and 0.19 μM/μg·day for Ca^2+^ and Si^4+^, respectively, into the outer environment [23]. Combining the results from our study, it was assumed that when MBNs–NH_2_ could be internalized in rDPSCs, the MBNs–NH_2_ could release Ca^2+^ and Si^4+^ and thereby orchestrate the fate of rDPSCs into odontoblasts. It has already been shown that Ca^2+^ and Si^4+^ combinatorially regulate dental originated stem cells to become odontoblasts and to undergo odontoblast differentiation [42,66].

### Differentiation of rDPSCs into odontoblasts

BSP and COL1A, which are well-known early markers of odontoblast differentiation [67], were significantly expressed in 50 μg/mL of MBNs–NH_2_ within 7 days, as compared with the OM-treated control. Other odontogenic markers at a relatively late stage such as DMP1, DSPP, and OCN were significantly expressed in the MBNs–NH_2_-treated groups from 14 or 21 days, as compared with the OM-treated control. We also examined ALP activity and ARS correlating with calcium-phosphate matrix formation in dentin prior to the initiation of mineralization [68]. High ALP activity provides high concentrations of phosphate at the site of mineral deposition. ARS is used to quantitatively determine the presence of calcific deposition by cells, which is a crucial step toward the formation of calcified extracellular matrix. Differentiated odontoblasts from DPSCs produce dentin, which consists of calcium and phosphate. Therefore, the above two assays were promising for the evaluation of odontogenic differentiation [9]. Significant levels of ALP and ARS were detected in the MBNs–NH_2_–treated cells, and treatment with 50 μg/mL had more ALP activity and relative mineralization than 25 μg/mL, which is consistent with the qPCR results from 14 days and 21 days. Therefore, it was concluded that MBNs–NH_2_ have concentration-dependent biological effects in terms of odontoblast differentiation in rDPSCs.

In previous experiments, osteogenic or odontogenic differentiation of stem cells was encouraged by dissolution ions (Si^4+^, Ca^2+^ and PO_4_^3-^) of bioactive glass as well as co-culture with bioactive glass [29,30,69,70]. The osteogenic differentiation was significantly enhanced in bioactive glass nanoparticle–encapsulated stem cells compared with bioactive glass nanoparticle–conditioned medium culture group, which means internalization of bioactive glass nanoparticle is able to help differentiation [71]. In addition, when stem cells were exposed to a pulse of bioactive glass nanoparticle for 24 hours and further incubated with fresh media to exclude additional ionic microenvironment from noninternalized particles, the cells were still differentiated into osteogenic lineage under differentiated media conditions, and ALP activity was significantly increased compared with the nontreated control [59].

Combining the above results, the internalization of bioactive glass nanoparticles into stem cells is required to rapidly differentiate into an osteogenic lineage from stem cells; consequently, odontogenic differentiation is able to be increased by endocytosis of bioactive glass nanoparticles owing to the similar hard tissue regenerative potential of osteogenic cells and odontogenic cells. When internalized into rDPSCs, MBNs–NH_2_ are considered to deliver odontoblastic differentiating ions such as Ca^2+^ and Si^4+^, and this expectation is confirmed by the results of ICP-AES in this study. Extracellular Ca^2+^ and Si^4+^ are considered to increase odontoblastic or osteoblastic differentiation, but the intracellular levels or effects of Ca^2+^ and Si^4+^ in differentiated odontoblasts have rarely been studied [72-74]. Increased intracellular Ca^2+^ concentrations from total cells under no significant difference in the total cell numbers was detected in the bioactive glass–treated osteoblasts, which contributed to the increased formation of hard tissue by the osteoblasts [75]. Extracellular Si^4+^ treatment from bioactive glass–conditioned media significantly increased the differentiation of osteoblasts even in the absence of osteogenic supplements [69]. By combining these results with the intracellular diffusion of Si^4+^, it can be postulated that increased intracellular Ca^2+^ and Si^4+^ concentrations from total cells also benefit odontoblastic differentiation. ICP-AES confirmed the significant increase in intracellular Ca^2+^ and Si^4+^ concentrations from total MBNs–NH_2_–treated rDPSCs compared with their untreated counterparts when there was no significant difference of total cell number between two groups at any time during incubation (4, 12, 24, 48 or 72 hours). Therefore, along with increased odontoblastic differentiation, elevated Ca^2+^ and Si^4+^ concentrations due to internalized MBNs–NH_2_ was confirmed and is considered to contribute odontoblastic differentiation. Further study is needed to clarify that the intracellular ions induced odontoblastic differentiation in the rDPSCs and related cellular mechanisms.

Beyond bioactive glass nanoparticles, other bioactive nanoparticles have been introduced in hard tissue engineering. Metal oxides such as titanium dioxide (TiO_2_) or magnesium oxide (MgO) nanoparticles were investigated for bone tissue engineering applications with regard to bioactivity, but they lack biodegradability [76,77]. As for biodegradable ceramic nanoparticles, calcium phosphate–based nanoparticles (e.g., hydroxyapatite, tricalcium phosphate, octacalcium phosphate, amorphous calcium phosphate, dicalcium phosphate, etc) are targeted for applications in regeneration of hard tissue with its compositional to human hard tissue, good biocompatibility, bioactivity, and osteoconductivity [78]. However, the ability to control calcium phosphate–based nanoparticle size and modify its morphology is limited by currently available fabrication processes [79]. Bioactive glass nanoparticles are considered as promising biomaterial used in the fields of dentistry and orthopedics. Along with their bioactivity, bioactive glass nanoparticles are known to be biocompatible and biodegradable without resulting in toxicity, inflammation, and a foreign-body response [80]. In addition, it is relatively easy to modify the morphology of bioactive glass nanoparticles (e.g., their mesoporosity) and to control their size by sol-gel methods [81]. Above characteristics are the reasons why bioactive glass nanoparticle was chosen for dentin regeneration in this study.

Direct comparisons of the above-mentioned nanoparticles in terms of bioactivity and consequent odontoblastic differentiation is beyond the scope of this discussion; however, compared with metal oxide nanoparticles, bioactive glass nanoparticles have increasingly shown bioactivity owing to their biodegradability [82]. Compared with calcium phosphate–based nanoparticles, bioactive glass nanoparticles have similar bioactivity and the potential for significantly enhancing their bioactivity by means of morphological tunability (e.g., a more mesoporous structure) with the development of the sol-gel processing method. For use in therapeutic dental materials, calcium phosphate–based nanoparticles have limited esthetic appeal because they transmit less light from the crystal structure. Because dental materials must be able to transmit a lot of light, the amorphous structure of the bioactive glass nanoparticles seems to be a promising characteristic, which is why we chose to study them with regard to odontoblastic differentiation. Future studies to directly compare calcium phosphate nanoparticles and bioactive glass nanoparticles in terms of their bioactivity and optical properties are still needed to optimize the use of nanoparticles for odontoblastic differentiation.

## Conclusions

MBNs–NH_2_ were developed as an additive material for the differentiation of rDPSCs into odontoblasts. A well-ordered mesoporous structure was maintained after amination, which successfully changed the zeta-potential from a negative to a positive charge. The *in vitro* biological effects in rDPSCs were investigated in terms of cell viability, uptake of MBNs–NH_2,_ and odontogenic differentiation. Cell viability was not changed during MBNs–NH_2_ treatments up to 50 μg/mL; uptake efficiency was calculated to be ≈92%, and ATP-dependent macropinocytosis was involved as the endocytosis pathway. Differentiation into odontoblasts was confirmed by ALP activity, ARS staining, and the mRNA levels of odontoblastic genes, including BSP, COL1A, DMP-1, DSPP, and OCN. Based on this work, MBNs–NH_2_ are potentially useful nano-additives for dental pulp tissue regenerative materials with biocompatibility and odontogenesis.

## Supporting Information

S1 File Supporting Information file for the results from BET and flow cytometry analysis. **Figure A.** Results of Brunauer–Emmett–Teller analysis showing typical mesoporous N_2_ adsorption–desorption curve. **Figure B.** Uptake of FITC-labeled MBNs-NH_2_ into rDPSCs within 4 hours with or without pre-treatment using various types of inhibitors or culture conditions. Incubation at 4°C for 4 hours was used to prevent ATP-dependent endocytosis. (**a**) Uptake as characterized by flow cytometry according to incubation time. (**b-g**) Uptake as characterized by flow cytometry according to pre-treatment condition (1 hour). Pre-treatments included 100 mM of sodium azide (SA), inhibitor of ATP-dependent endocytosis; 2.5 mM of 5-(N-ethyl-N-isopropyl) amiloride (AE), inhibitor of macropinocytosis; 1 mM of amantadine-HCl (AT), inhibitor of clathrin-mediated endocytosis; and 100 mM of genistein (GE), inhibitor of caveolae-mediated endocytosis.
(PDF)
