# Supplementary material for: Effect of Aminated Mesoporous Bioactive Glass Nanoparticles on the Differentiation of Dental Pulp Stem Cells
Source: PLoS One. 2016 Mar 14;11(3):e0150727. doi: 10.1371/journal.pone.0150727 (PMC4790939; doi:10.1371/journal.pone.0150727)
Supplement: S1 File — Figure A. Results of Brunauer–Emmett–Teller analysis showing typical mesoporous N2 adsorption–desorption curve. Figure B. Uptake of FITC-labeled MBNs-NH2 into rDPSCs within 4 hours with or without pre-treatment using various types of inhibitors or culture conditions. Incubation at 4°C for 4 hours was used to prevent ATP-dependent endocytosis. (a) Uptake as characterized by flow cytometry according to incubation time. (b-g) Uptake as characterized by flow cytometry according to pre-treatment condition (1 hour). Pre-treatments included 100 mM of sodium azide (SA), inhibitor of ATP-dependent endocytosis; 2.5 mM of 5-(N-ethyl-N-isopropyl) amiloride (AE), inhibitor of macropinocytosis; 1 mM of amantadine-HCl (AT), inhibitor of clathrin-mediated endocytosis; and 100 mM of genistein (GE), inhibitor of caveolae-mediated endocytosis. (PDF) [file pone.0150727.s001.pdf]

Supporting Information file (S1) for the results from BET and flow cytometry analysis.

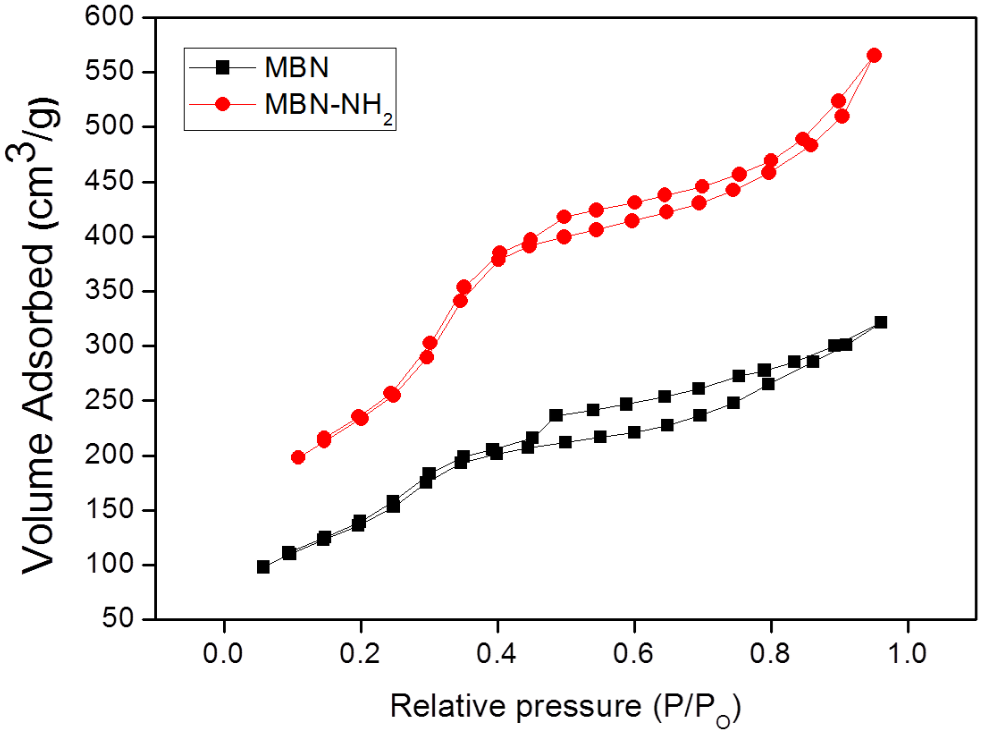

Figure A. Results of Brunauer–Emmett–Teller analysis showing typical mesoporous N_2 adsorption–desorption curve.

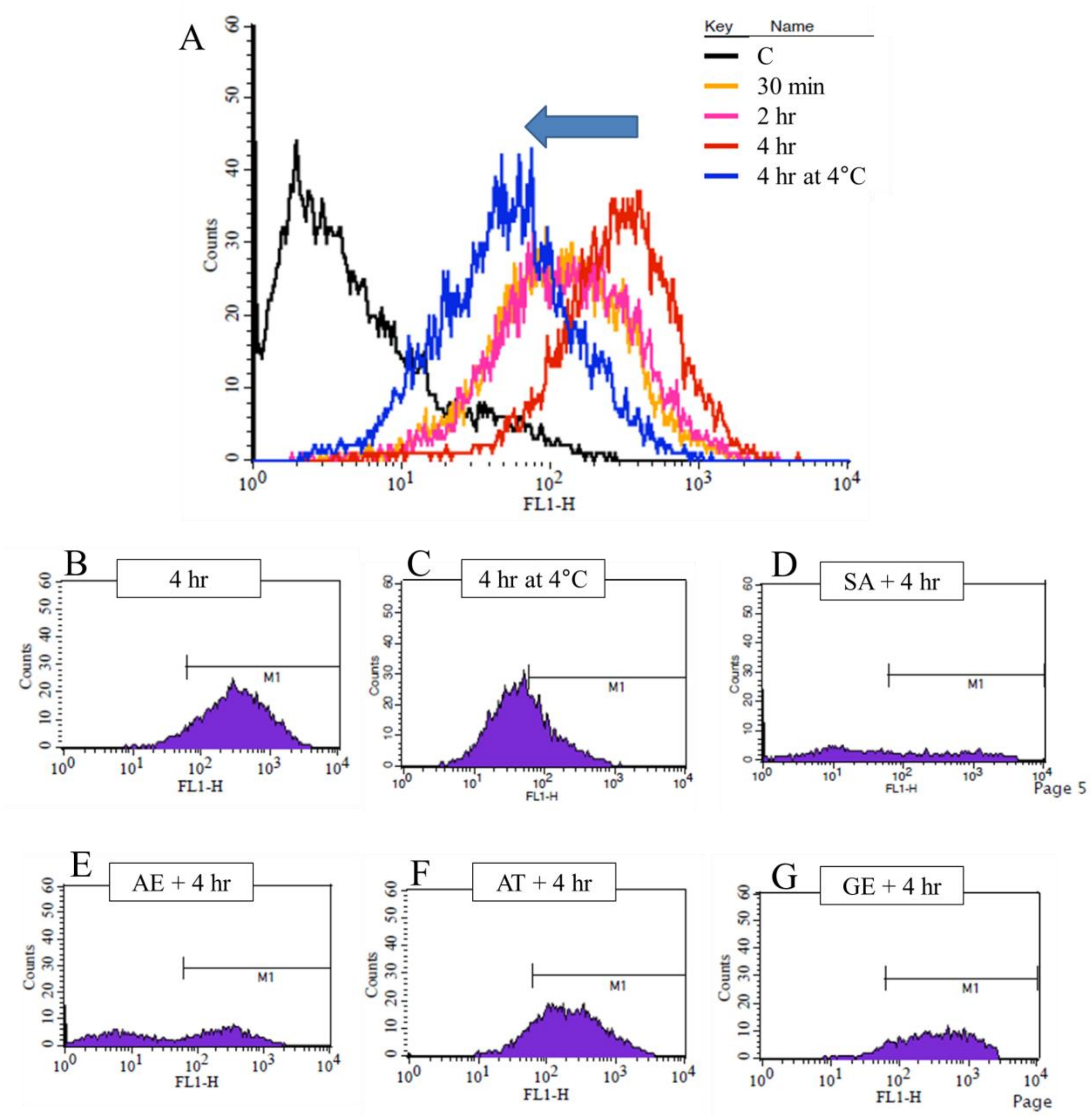

Figure B. Uptake of FITC-labeled MBNs-NH_2 into rDPSCs within 4 hours with or without pre-treatment using various types of inhibitors or culture conditions. Incubation at 4℃ for 4 hours was used to prevent ATP-dependent endocytosis. (**a**) Uptake as characterized by flow cytometry according to incubation time. (**b-g**) Uptake as characterized by flow cytometry according to pre-treatment condition (1 hour). Pre-treatments included 100 mM of sodium azide (SA), inhibitor of ATP-dependent endocytosis; 2.5 mM of 5-(N-ethyl-N-isopropyl) amiloride (AE), inhibitor of macropinocytosis; 1 mM of amantadine-HCl (AT), inhibitor of clathrin-mediated endocytosis; and 100 mM of genistein (GE), inhibitor of caveolae-mediated endocytosis.
